# Dimeric Bis-Benzimidazole-Pyrroles DB2Py(n) – AT-Site-Specific Ligands: Synthesis, Physicochemical Analysis, and Biological Activity

**DOI:** 10.32607/actanaturae.27327

**Published:** 2024

**Authors:** O. Y. Susova, S. S. Karshieva, A. A. Kostyukov, N. I. Moiseeva, E. A. Zaytseva, K. V. Kalabina, E. Zusinaite, K. Gildemann, N. M. Smirnov, A. F. Arutyunyan, A. L. Zhuze

**Affiliations:** N.N. Blokhin National Medical Research Center of Oncology of the Ministry of Health, Moscow, 115522 Russian Federation; Emanuel Institute of Biochemical Physics, Russian Academy of Sciences, Moscow, 119334 Russian Federation; Engelhardt Institute of Molecular Biology, Russian Academy of Sciences, Moscow, 119991 Russian Federation; Tartu University Institute of Technology, Tartu, 50411 Estonia

**Keywords:** bis-benzimidazole-pyrrole, DNA narrow groove binding ligand, cytotoxicity, DNA-binding, topoisomerase I, cell cycle, multidrug resistance

## Abstract

Its broad spectrum of biological activity makes benzimidazole a fundamental pharmacophore in pharmaceutics. The paper describes newly synthesized AT-specific fluorescent bis-benzimidazole molecules DB2Py(n) that contain a pyrrolcarboxamide fragment of the antibiotic drug netropsin. Physico-chemical methods using absorption, fluorescence, and circular dichroism spectra have shown the ability of bis-benzimidazole- pyrroles to form complexes with DNA. The new DB2Py(n) series have turned out to be more toxic to human tumor lines and less vulnerable to non-tumor cell lines. Bis-benzimidazole-pyrroles penetrated the cell nucleus, affected the cell-cycle synthesis (S) phase, and inhibited eukaryotic topoisomerase I in a cellfree model at low concentrations. A real-time tumor cell proliferation test confirmed the molecule’s enhanced toxic properties upon dimerization. Preliminary cytotoxicity data for the bis-benzimidazole-pyrroles tested in a cell model with a MDR phenotype showed that monomeric compounds can overcome MDR, while dimerization weakens this ability to its intermediate values as compared to doxorubicin. In this respect, the newly synthesized cytotoxic structures seem promising for further, in-depth study of their properties and action mechanism in relation to human tumor cells, as well as for designing new AT-specific ligands.

## INTRODUCTION

Compounds capable of effectively binding to a narrow DNA groove by forming hydrogen bonds are of interest as agents that can help regulate biological activity. In this respect, the use of bis-benzimidazoles as drugs opens new opportunities for the therapy of socially significant diseases, including malignant neoplasms. The rapidly growing tumor resistance to existing treatment protocols makes it necessary to accelerate the search for new effective DNA-specific ligands, which now represent an important direction in the development of medicinal chemistry [1, 2, 3, 4].

DNA-specific compounds based on DNA narrowgroove- binding ligands target the AT pairs of nucleotides in the DNA structure. It is possible to match a molecular structure to a preselected binding site on the DNA, as well as to eliminate any nonspecific interaction with the DNA through complementary interactions between a DNA-specific ligand and a biomacromolecule. In this regard, DNA narrow-groove-binding ligands, which are compounds of low molecular weight interacting noncovalently and sitespecific with DNA, seem to be quite promising. Such compounds are largely free of the adverse mutagenic side effects characteristic of the low-molecular-weight compounds that intercalate between DNA base pairs. They are able to modulate the expression of genes and DNA-binding proteins, thus exhibiting anti-tumor properties.

Earlier, we obtained **Hoechst 33258**-based fluorescent water-soluble AT-specific dimeric bis-benzimidazole DNA narrow-groove-binding ligands of the** DB(n)**, **DBP(n)**, **DBA(n), **and **DBPA(n) **series, where n is equal to the number of methylene links in the linker. All the above-mentioned compounds contained two AT-recognizing fragments in their structure, consisting of two bis-benzimidazole units [5, 6, 7, 8]. Upon interaction with DNA, each AT-recognizing fragment formed a bifurcation (three-center) hydrogen bond with an O_2_ thymine atom and/or an adenine atom of two neighboring AT pairs, while covering a region of approximately one and a half base pairs [9]. All the series penetrated cellular and nuclear membranes, stained DNA, and showed significant activity as inhibitors of DNA-dependent enzymes.

**Fig. 1:**
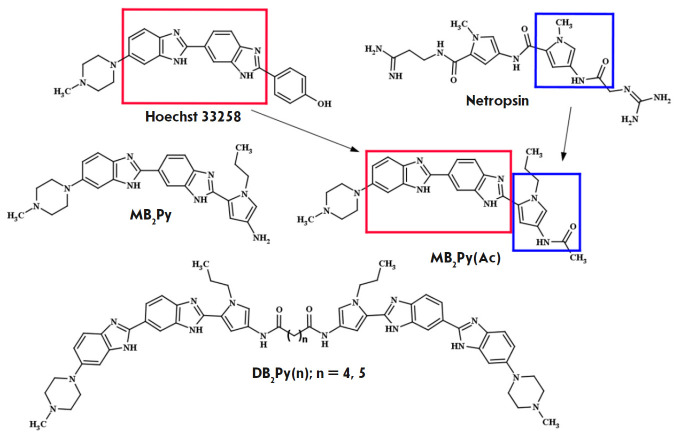
Structural formulas of **Hoechst 33258**, netropsin, monomeric **MB2Py**, **MB2Py(Ac), **and dimeric compounds** DB2Py(n)**. In **MB2Py(Ac)**, the bis-benzimidazole fragment is highlighted in red; and the pyrrolecarboxamide fragment, in blue

In the present study, we synthesized and investigated the biological activity of new DNA narrowgroove- binding ligands containing in their structure an AT-recognizing pyrrolcarboxamide fragment similar to that of netropsin [10], a natural antibiotic never used in practice due to its high cytotoxicity. The new ligands – **MB2Py **and **MB2Py(Ac)** (*Fig. 1*) – consist of three (two benzimidazole and one pyrrolcarboxamide) AT-recognizing units covalently linked to each other, and the dimeric derivatives **DB2Py(n)** (*Fig. 1*) dimerized from **MB2Py **by oligomethylene α,ω-dicarboxylic acids of various lengths (**n **here is the number of methylene links in the linker) to form symmetric head-to-head type compounds. The flexible linker in dimeric compounds allows the molecule to bind to two AT-rich sites located at different distances from each other. As we showed earlier, dimerization of the monomeric ligand of **DB(n) **series increased affinity to the newly structured DNA [11].

Increasing the number of AT-recognizing fragments in the monomeric subunit increases the ligand DNA complexation constant and should decrease the inhibitory concentration against DNA-dependent enzymes. The paper describes the synthesis of two monomeric compounds, **MB2Py **and **MB2Py(Ac)**, and the dimeric compounds **DB2Py(n) **and investigates their biological activity.

**Fig. 2:**
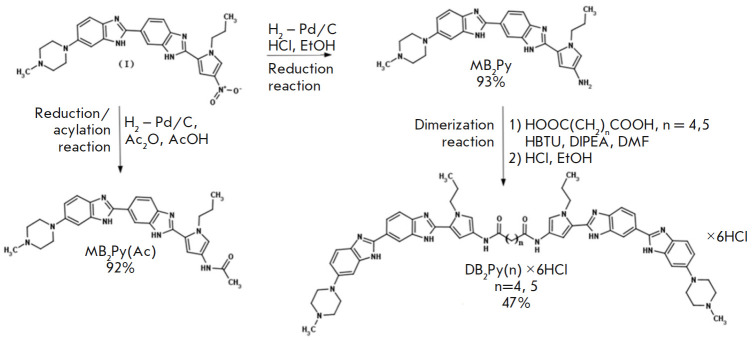
Synthesis scheme of monomeric (**MB2Py, MB2Py(Ac)**) and dimeric (**DB2Py(4, 5)**) compounds

The dimeric bis-benzimidazole-pyrroles,** DB2Py(4, 5), **were synthesized from 6-[6-(4-methylpiperazin- 1-yl)-1H-1,3-benzodiazol-2-yl]-2-(4-nitro- 1-propyl-1H-pyrrol-2-yl)-1H-1,3-benzodiazole **(I)** (*Fig. 2*) obtained in the Laboratory of DNA-Protein Interactions at the Institute of Molecular Biology of the Russian Academy of Sciences. Compound **(I) **was used to obtain the monomeric ligand **MB2Py(Ac) **by reducing the parent compound in a hydrogen current in glacial acetic acid in the presence of acetic anhydride on 10% palladium with carbon as a catalyst. A monomeric unit of **MB2Py **was dimerized into two dimeric compounds **DB2Py(n) **with a series of aliphatic normal α,ω-dicarboxylic acids in the presence of HBTU as a condensing agent and DIPEA as a DMF base, differing in the number of methylene links in the linker: *n *= 4, 5 (*Fig. 2*).

## EXPERIMENTAL

In this study, such chemical substances as adipic and pimelic acids, HBTU, DIPEA (Fluka, Switzerland), dioxane, DMF, ice-cold AcOH, Ac2O, iPrOH, acetone (Reachim, Russia) were used. Solutions in organic solvents were dried over Na_2_SO_4_. The solvents were evaporated on a rotary evaporator in the vacuum of a water jet pump, usually at 30–50°C. The substances were dried in vacuo over P2O5 and NaOH. The melting temperatures were determined with a Boethius device (Germany). Hydrogenation was carried out over 10% Pd/C (Merck, Germany) at atmospheric pressure and room temperature until hydrogen absorption ceased. The purity of the obtained compounds was determined by TCX on Kieselgel 60 F254 plates (Merck). Substances in chromatograms were detected in UV light by absorbance at 254 nm and/or fluorescence at 365 nm.

**1H-NMR spectra **were recorded using an Avance III 300 MHz spectrometer (Bruker, Germany) equipped with a cryogenic TCI triple resonance probe (Bruker Biospin, Gmbh, Germany) in DMSO-*d*6 at 30°C.

**Mass spectra **were taken using an AB SCIEX 4800 TOF analyzer (AB SCIEX, USA) in positive-ion detection mode (unless otherwise specifically stated); 2,5-dihydroxybenzoic acid matrix; N2–laser of 337 nm.

**Absorption spectra **were recorded in a Cary100 spectrophotometer (Varian, USA).

**Fluorescence spectra **of the solutions were measured in a PTI spectrofluorimeter (Photo Technology Intern., Canada) using double-stranded DNA of calf thymus (Sigma).

**CD spectra **were recorded in a portable SKD-2 dichrometer (Institute of Spectroscopy, Russian Academy of Sciences, Troitsk) using DNA of salmon sperm (Technomedservice, Russia) and quartz cuvettes with an optical path length of 1 cm.

**Human tumor cell lines **were investigated using human non-small cell lung cancer cell lines A549, colon cancer HCT-116, hepatocarcinoma Huh7, pancreatic carcinoma PANC-1, breast cancer SKBR3, MCF7, ovarian cancer SKOV3, osteosarcoma U2OS, a primary culture of human glioblastoma Gbl13n, immortalized epithelial cell line HBL-100 and its doxorubicin- resistant subline HBL-100/DOX, and breast cell line MCF10A of non-tumor origin. As control drugs irinotecan, doxorubicin (Sigma), etoposide, and puromycin (InvivoGen) were applied.

For human tumor cell lines, the DMEM medium (Sigma) containing 10% fetal calf serum (HyClone, South America) and 2 mM *L*-glutamine (PanEco, Russia) was used. Non-tumorigenic MCF10A cells were cultured in a DMEM/F12 medium (Sigma) containing 5% horse serum (Biosera, South America), 100 mg/mL EGF, 1 mg/mL hydrocortisone, and 10 mg/mL insulin (PanEco). Both media contained 100 U/mL penicillin and streptomycin (PanEco), and all cells were cultured at 37°C, 5% CO_2_.

**The cytotoxic effect **was evaluated using the standard microculture tetrazolium test (MTT) measuring the ability of live cell dehydrogenases to reduce uncoated forms of 3-(4,5-dimethylthiazol-2-yl)-2,5-diphenyl- tetrazolium bromide (PanEco) to the blue crystalline pharmasan soluble in dimethyl sulfoxide (DMSO). The coloration was recorded at a wavelength of 570 nm using a spectrophotometer (Multiskan FC, Thermo Fisher Scientific, USA). The optical density in the wells with the cells incubated without the drug was taken as 100%. The optical density values in the wells with each control concentration were averaged, and the percentage of surviving cells for a particular drug concentration was calculated.

**Cell cycle measurements **were performed using flow cytofluorimetry. To do so, HCT-116 cells were seeded at 500 × 10^3^ cells per well in a 6-well plate and grown in the DMEM medium (Gibco, USA) containing 2 mM* L*-glutamine (PanEco) and a 1X antibiotic-antimycotic solution (Gibco) supplemented with 10% (v/v) fetal bovine serum (Gibco) at 37°C and 5% CO_2_. In 24 h, the medium was changed to fresh and drugs added at a concentration of 1 μM with no drug added to the control wells. The cells were incubated for 24 and 48 h to be washed in dishes with the Versen (PanEco) and 0.25% trypsin/EDTA(Gibco) solutions. The cell concentration was counted, and equal amounts were selected for the analysis. The selected cells were centrifuged for 5 min at 1,200 rpm to remove the supernatant and washed in 1 ml of cold PBS. The cells were resuspended in 1 ml of cold 96% ethanol and incubated overnight at +4°C to be centrifuged (15 min, 1,900 rpm) and washed 1 time with a cold PBS solution. The precipitate was added, 1 ml of the 3.8 mM sodium citrate solution in PBS containing 500 μg/ml propidium iodide and 1 μl of RNase A (10 mg/ml), and incubated overnight at +4°C. Propidium iodide fluorescence was measured in the FL2 channel using a CytoFlex flow cytofluorimeter. The obtained data were analyzed using the ModFit LT 3.2 software (Verity Software).

**Proliferation of the cells **exposed to the tested compounds was determined using xCELLigence RTCA (ACEA Biosciences, USA). Every well of a 16-well plate was seeded with 5,000 human osteosarcoma U2OS cells. Each well of the plate had a microelectronic biosensor at the bottom (proprietary E-plates). The cells were incubated for 24 h in a Roche xCELLigence RTCA DP (Roche Diagnostics GmbH, Germany). When cell index 1 was reached, the medium was removed to be added either to the same medium (control) or media with different substance concentrations (0.16, 0.8, 4, 20, 100, 500 μM, respectively).

To determine **the ability of the compounds to penetrate the cell nucleus, **live Gbl13n glioblastoma cells were incubated in the culture medium with the compounds at a concentration of 2 μM for 2 days, washed with phosphate-salt buffer, fixed with formaldehyde, and photographed using a Zeiss Axioplan 2 fluorescence microscope (Carl Zeiss, Germany) in the ultraviolet wavelength range.

**Inhibition of Topo-I catalytic activity **was assessed in a relaxation reaction of supercoiled DNA (scDNA). The ability to modulate Topo-I activity *in vitro *was studied using a Topoisomerase I Drug Screening kit (TopoGen, Inc., cat. no. 1018-1, www.topogen.com). A single unit of purified Topo-I from calf thymus (Fermentas, USA) and the tested compounds at concentrations of 2.5 and 5 μM; 0.65 and 1.25 μM were incubated with 0. 12 μg of pHOT1 supercoiled plasmid DNA (TopoGen) in ×1 reaction buffer (10 mM Tris-HCl pH 7.9, 1 mM EDTA, 0.15 M NaCl, 0.1% BSA, 0.1 mM spermidine, 5% glycerol). The reaction was run for 30 min at 37°C, stopped by adding SDS to a final concentration of 1%, and treated with proteinase K of final concentration of 50 μg/mL for 30–0 min at 37°C. The reaction products were separated electrophoretically in a 1% agarose gel with TAE buffer (2 M Tris base, 0.05 M EDTA, 1.56 M acetic acid) at a maximum electric field strength of 3–4 V/cm and then stained with an aqueous ethidium bromide solution (0.5 μg/mL). DNA visualization in gel was performed by fluorescence in transmitted ultraviolet light at wavelengths ranging from 240 to 360 nm. In the absence of the inhibitor, Topo-I relaxed scDNA to form a series of topoisomers. Topo-I inhibition effect was detected by the ability of the tested compounds to delay the relaxation reaction of scDNA; i.e., by its preservation. The initial concentration of bis-benzimidazole- pyrroles in DMSO was 5 × 10-3 M.

**Cell survival data **were processed in the GraphPad Prism 8 software, and the viability curves were compared using Fisher’s criterion (F-test). All experiments were repeated 3 times, and the effect on cell proliferation in real time was tested twice. For the purpose of data presentation, the most successful experiment, whose results did not contradict those of the same experiments, was selected.

## RESULTS

**Synthesis of monomeric bis-benzimidazolepyrroles MB2Py and MB2Py(Ac), and dimeric compounds DB2Py(4, 5)**

*MB2Py synthesis. *0.1 g of 10% Pd/C suspended in 20 mL of absolute ethanol was saturated with hydrogen until its absorption ceased. Then, 0.3 mL of concentrated hydrochloric acid and 0.20 g (0.41 mmol) of 6-[6-(4-methylpiperazin-1-yl)-1H-1,3-benzodiazol- 2-yl]-2-(4-nitro-1-propyl-1H-pyrrole-2-yl)-1H-1,3-benzodiazole** (I) **was added. The reaction mixture was stirred at room temperature until hydrogen absorption ceased. The resulting solution was filtered off the catalyst and precipitate, the solid precipitate of the target substance was washed off the filter with 2 × 10 mL water, and the water was evaporated under reduced pressure. The **MB2Py **yield was a green amorphous powder weighing 0.173 g (93%). TLC analysis in a hexane/ethyl acetate solvent system (3 : 1) showed that the obtained substance was homogeneous. Its mass spectrum was 455.26 [M+H]+ and 454.57 (C26H30N8) for the calculated one.

*MB2Py(Ac) synthesis**. ***0.15 g of 10% Pd/C suspended in 30 mL of glacial acetic acid was saturated with hydrogen until its absorption ceased. Then, 2 mL of acetic anhydride and 0.35 g (0.72 mmol) of the substance** (XII) **were added. The reaction mixture was stirred in a room-temperature hydrogen current for 5 h. The resulting solution was filtered off the catalyst. The resulting solution was evaporated under reduced pressure to be redissolved three times with 30 mL of isopropyl alcohol. The **MB2Py(Ac) **yield was 0.37 g (92.4%) of yellow crystals. TLC analysis in a* i-*PrOH–NH_4_OH (5:1) solvent system showed that the obtained substance was homogeneous; and its melting temperature – 219°C. Its mass spectrum was m/z: 496.15 [M]+, 454.13 [M–NHAc]+, and 496.26 (C28H32N8O) for the calculated one.

*General synthesis of DB2Py(n). A *α,ω-alkyldicarboxylic acid (0.1 mmol) solution in 2 mL of abs DMF was added to HBTU (0.25 mM), DIPEA (0.50 mM) and stirred at room temperature for 30 min. The resulting solution was added to 0.10 g (0.2 mmol) of MB2Py, stirred for another hour, and the reaction mixture was left overnight. The solvent was evaporated under reduced pressure, the resulting oil was mashed with abs* i*-PrOH to add 0.5 mL of 35% HCl in dioxane, and the precipitate was filtered off, washed 3 times with 80% aqueous acetone and 2 times with abs *i*-PrOH. The solid residue as a green powder was dried in vacuo over NaOH/P2O5. TLC analysis in a MeOH-TFA-H_2_O (5:1:2) solvent system showed that the obtained substance was homogeneous.

**DB2Py(4)**•E6HCl. Yield 55 mg (48%), melt. temp. > 350°C. Mass spectrum: 1019.57 [M+H]+, calculated Ms: 1018.55 (C58H66N16O_2_). 1H-NMR (300 MHz, DMSO-*d*6): *δ *0.85 (6H, t, *J *= 7.4, 2(–C**H3**)), 1.65 (4H, m, (–C**H2**–C**H2**–)), 1.75 (4H, q, *J *= 7.2, 2(–CH_2_C**H2**CH_3_)), 2.31 (4H, m, 2(–COC**H2**–)), 2.77 (4H, s, pip), 3.23 (4H, s, pip), 3.35 (6H, s, 2(N–C**H3**)), 4.54 (4H, t, *J *= 7.0, 2(N–C**H2**–)), 7.05–6.84 (4H, m, ArH), 7.10 (2H, s, ArH), 7.36–7.24 (2H, brs, ArH), 7.48 (2H, d, *J *= 8.6, ArH), 7.58 (2H, m, ArH), 7.97 (2H, m, ArH), 8.26 (2H, d, *J *= 36.2, ArH), 9.93 (2H, s, 2(–N**H**CO–)).** DB2Py**(**5)**•E6HCl. Yeild 61 mg (47%), melt. temp. > 350°C. Mass spectrum: m/z: 1033.42 [M+H]+, calculated Ms: 1032.57 (C59H68N16O_2_). 1H-NMR (300 MHz, DMSO-*d6*): *δ *0.87 (6H, t, *J *= 7.4, 2(–C**H3**)), 1.34 (2H, m, –C**H2**–), 1.63 (4H, m, –C**H2**–CH_2_–C**H2**–), 1.77 (4H, q, *J *= 7.2, 2(–CH_2_C**H2**CH_3_)), 2.30 (4H, m, 2(–COC**H2**–)), 2.87 (8H, brs, pip), 3.35 (6H, s, (–NC**H3**)), 4.55 (4H, t,* J *= 7.2, N–C**H2**–), 7.05 (2H, brs, ArH), 7.24 (2H, m, ArH), 7.42–7.28 (4H, m, ArH), 7.75 (4H, dd, *J *= 20.0, 8.6, ArH), 8.04 (2H, d, *J *= 8.7, ArH), 8.45 (2H, brs, ArH), 9.93 (2H, s, 2(–N**H**CO–)).

## PHYSICO-CHEMICAL ACTIVITY

**DB_2_Py(n) absorption and fluorescence spectra**

**Fig. 3:**
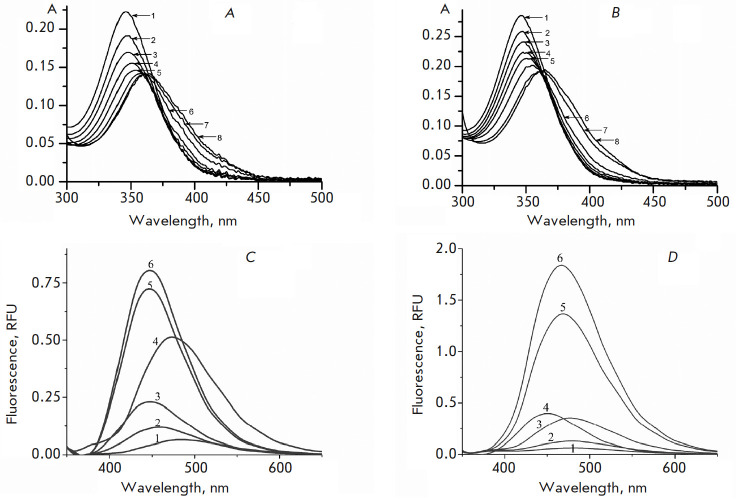
Absorption and fluorescence spectra of free **DB2Py(n) **and its complex with DNA. (*A*) – absorption spectra of **DB2Py(4) **in absence (*1*) and presence of DNA (*2–8*); [**DB2Py(4)**] 4.06 × 10-6 M; 0.001 M sodium cacodylate. [DNA] *1 *– 0; *2 *– 0.25; *3 *– 0.49; *4 *– 0.98; *5 *– 1.48; *6 *– 2.45; *7 *– 14.67; *8 *– 121.9 × 10-6 M b.p. respectively; optical path length, 10 mm. (*B*) – absorption spectra of **DB2Py(5) **in absence (*1*) and presence of DNA (*2*–*8*); [**DB2Py(5)**] 4.29 × 10-6 M; 0.001 M sodium cacodylate. [DNA] *1 *– 0; *2 *– 0.25; *3 *– 0.49; *4 *– 0.98; *5 *– 1.48; *6 *– 2.45; *7 *– 14.67; *8 *– 121.9 × 10-6 M b.p. respectively; optical path length, 10 mm. (*C*) – fluorescence spectra of **DB2Py**(**4**) in absence *(1) *and presence of DNA (*2*–6). [**DB2Py**(**4**)] 4.6 × 10-6 M; [DNA]* 1 *– 0; *2 *– 3; *3 *– 6; 4 – 18; *5 *– 30; *6 *– 54 × 10-6 M b.p. respectively. Buffer: 10 mM PBS (pH 7.4). Excitation wavelength, 320 nm; slot width, 5 nm; cell size 10 × 10 mm; 22°C. (*D*) – fluorescence spectra of **DB2Py**(**5**) in absence *(1) *and presence of DNA (*2*–6). [**DB2Py**(**5**)] 2.3 × 10-6 M; [DNA]* 1 *– 0; *2 *– 3; *3 *– 6; *4 *– 18; *5 *– 54; *6 *– 78 × 10-6 M b.p. respectively. Buffer: 10 mM PBS (pH 7.4). Excitation wavelength, 320 nm; slot width, 5 nm; cell size 10 × 10 mm; 22°C

Measuring the intensity and absorption maxima of** DB2Py(4**) and **DB2Py(5**) in the absence and presence of DNA at different concentrations and comparison of the obtained spectra enabled us to indirectly confirm the ability of the new dimeric narrow-bridged ligands to form complexes with DNA (*Fig. 3 A,B*). As the DNA concentration increased, a drop in the absorption intensity was observed, indicating that the new bis-benzimidazole- pyrroles had formed a complex with DNA. Further increase in the DNA concentration led to a change in the absorption maximum position characterized by a shift to the long-wavelength region of the spectrum (bathochromic shift), as well as an increase in the amplitude of the absorption band. All these processes were indication that several types of complexes had formed depending on the ligand concentration.

In the presence of DNA, the fluorescence spectra of **DB2Py(4**) and **DB2Py(5**) showed increasing fluorescence intensity that grew together with the DNA concentration (*Fig. 3 C,D*). This was another sign that the compounds formed complexes with DNA, causing fluorescence ignition through stabilization of the conjugated ligand structure in the narrow DNA groove.

**CD spectra of DB2Py(4) and DB2Py(5)/DNA CLCD complexes**

The spectra allowed us to confirm that the obtained compounds formed complexes with DNA and detect their localization in one of the DNA grooves.

**Fig. 4:**
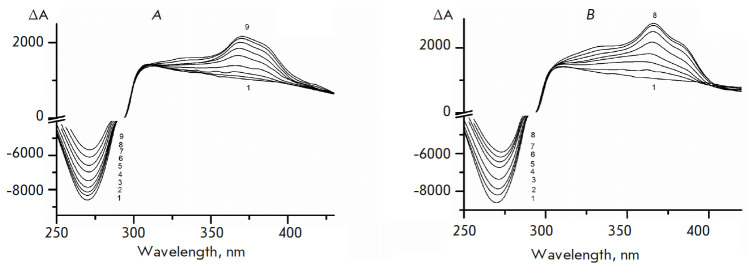
(*A*) – CD spectra in absence (*1*) and presence of (*2*–*9*) of **DB2Py(4)**; 0.3 M NaCl + 0.002 M Naphosphate buffer, pH 6.85; 170 mg/ml PEG-4000; [DNA] 4.545 × 10-5 M b*.*p.; [**DB2Py(4)**] *1 *– 0; *2 *– 0.41; *3 *– 0.82; *4 *– 1.63; *5 *– 3.25;* 6 *– 4.87; *7 *– 6.48; *8 *– 8.08; *9 *– 10.07 × 10-6 M respectively. Optical path length, 10 mm. (*B*) – CD spectra in absence (*1*) and presence of (*2*–*8*) **DB2Py(5)**; 0.3 M NaCl + 0.002 M Naphosphate buffer, pH 6.85, 170 mg/ml PEG-4000; [DNA] 4.545 × 10-5 M b.p.; [**DB2Py(5)**] *1 *– 0; *2 *– 0.43; *3 *– 0.86; *4 *– 1.72; *5 *– 3.44; *6 *– 5.15;* 7 *– 6.85; *8 *– 8.54 × 10-6 M respectively. Optical path length, 10 mm

A similar pattern was observed for compounds** DB2Py(4) **and **DB2Py(5)** (*Fig. 4*): a positive, intense band was detected in the ligand absorption region (300–400 nm), indicating the complexes localized in one of the DNA grooves [12, 13]. Since the X-ray diffraction analysis of the **Hoechst 33258 **parent compound localized it in the narrow groove of DNA [9], we had confirmation that our compounds are DNA narrow-groove-binding ligands.

## BIOLOGICAL ACTIVITY

**Cytotoxicity against human tumor cells**

**Fig. 5:**
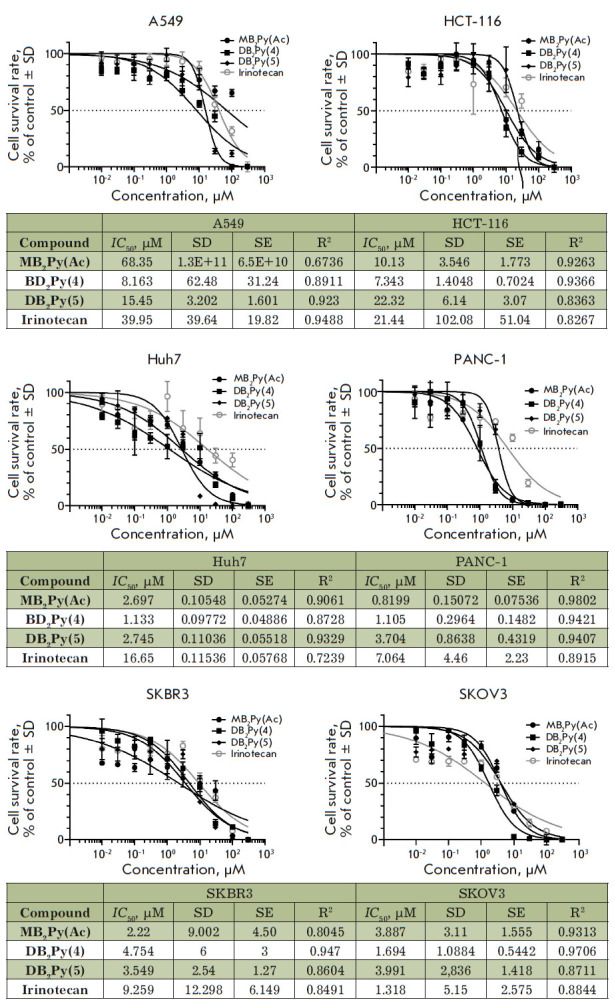
Cytotoxicity values (*IC*50) in micromoles (μM) of the monomeric and dimeric bis-benzimidazole- pyrroles compared to irinotecan in various human tumor cell lines. SD – standard deviation, SE – standard error, R2 – determination coefficient

The cytotoxic activity of the compounds was evaluated by the MTT method on six cell lines and one primary human tumor culture to determine the semi-inhibitory concentration (*IC*50) for non-small cell lung cancer A549, colon cancer HCT-116, hepatocarcinoma Huh7, pancreatic carcinoma PANC-1, breast cancer SKBR3, ovarian cancer SKOV3, and a primary culture of human glioblastoma Gbl13n we had obtained earlier [14, 15]. The data presented in *Fig. 5* demonstrate that the Huh7, PANC-1, and SKBR3 cell lines were more sensitive to the new compounds than to the antitumor agent irinotecan. The cytotoxicity of the dimeric molecules **DB2Py(4) **and **DB2Py(5) **against cell line A549 was significantly higher (5- to 7-fold) than that of monomeric **MB2Py(Ac) **and irinotecan (2.8- to 3.8-fold). However, no significant differences in the cytotoxicity of monomeric and dimeric bis-benzimidazole- pyrroles against the Huh7, PANC-2, SKBR3, and SKOV3 lines were detected.

Some believe that new drugs should be tested not only on linear, but also on primary cell cultures. In Gbl13n, a primary human glioblastoma cell culture, the cytotoxic activity of the **DB2Py(4)**, **DB2Py(5) **dimers was approximately 10-fold higher than that of the **MB2Py(Ac) **monomer and comparable to that of irinotecan (*Table 1*).

**Tumor-cell selectivity**

The possible selectivity of the new compounds against tumor cells was determined by the level of their cytotoxicity in tumor and the transformed cell lines.

**Table 1:** Cytotoxicity of monomeric and dimeric bis-benzimidazole-pyrroles versus irinotecan in a primary culture of human glioblastoma Gbl13n cells

Compound	IC_50_, μM
MB_2_Py(Ac)	> 100
DB_2_Py(4)	12.67 ± 2.33
DB_2_Py(5)	8.78 ± 6.64
Irinotecan	10.02 ± 0.7

**Fig. 6:**
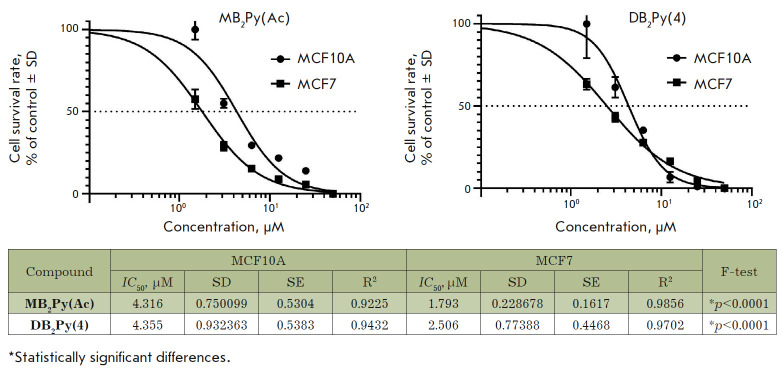
Cytotoxicity of the new bis-benzimidazole-pyrroles in MCF10A (human breast epithelial cells, normal cell line) and on MCF-7 (breast cancer). SD – standard deviation, SE – standard error, R2 – coefficient of determination, F-test – statistical criterion

Breast cancer (MCF7) and conditionally normal mammary epithelial (MCF10A) cell lines were used as models. The tested cell lines were sensitive to the toxic effects of new bis-benzimidazole-pyrroles, with MCF7 being the most susceptible (*Fig. 6*). Therefore, some, approximately twofold, selectivity in the cytotoxic action was observed. At the same time, the monomer **MB2Py(Ac) **and dimer **DB2Py(4) **exhibited similar *IC*50 values for the tested cell lines; so, it can be considered that the doubling of the molecule did not affect cytotoxicity against the investigated pair of cell lines.

**Proliferation in an osteosarcoma cell line**

**Fig. 7:**
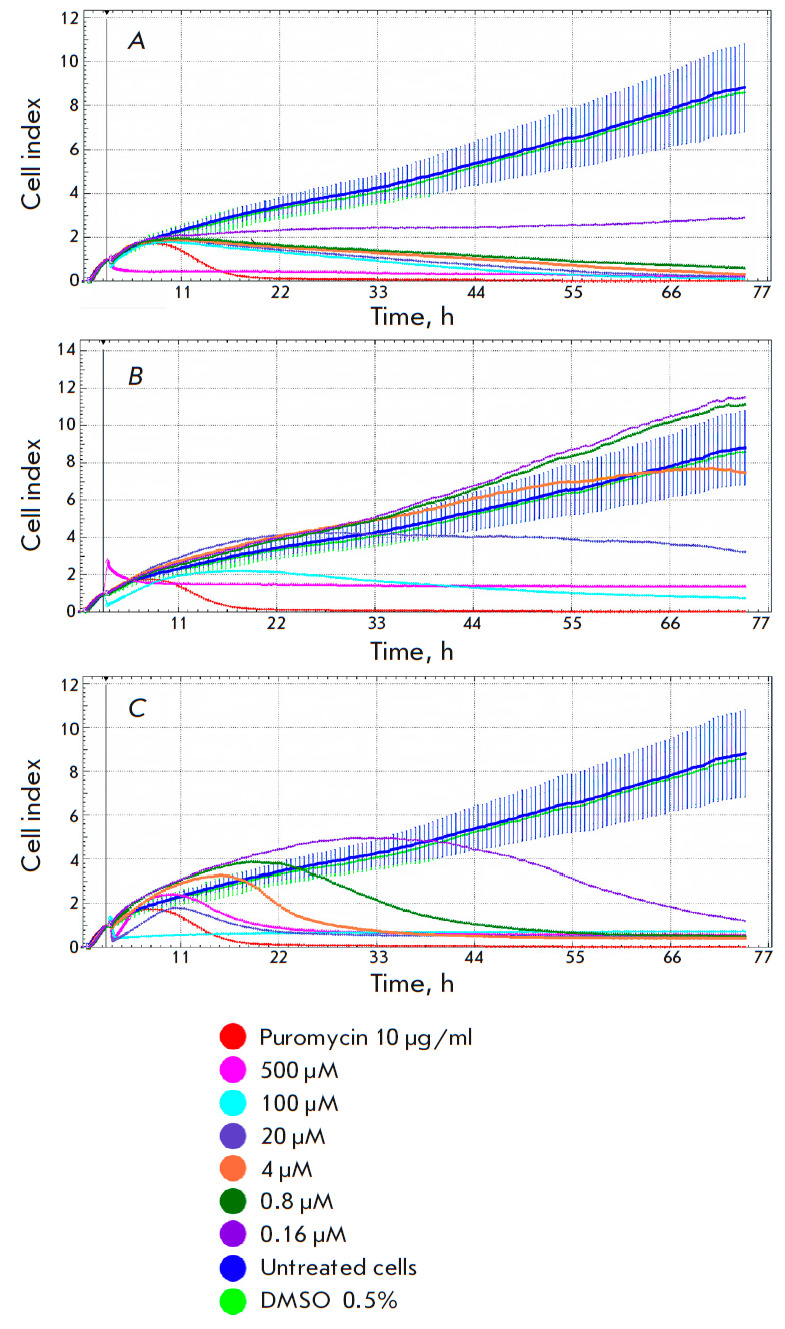
The effect of the bis-benzimidazoles **MB2Py(Ac)** and **DB2Py(5) **on the proliferation of osteosarcoma cells in real time. Growth curves were measured as the cell index over time. (*A*) – controls: puromycin 10 μg/ml (red line); growth of unexposed U2OS cell line (blue line); 0.5% DMSO (greenline). (*B*) – **MB2Py **exposure; (*C*) – **DB2Py(5)** exposure

**Proliferation in an osteosarcoma cell line** The effects the **MB2Py(Ac) **monomer and **DB2Py(5)** dimer had on the proliferation of the cultured U2OS osteosarcoma cells were compared in real time using an RTCA. After the compounds were introduced at concentrations of 0.16–500 μM, cell growth was recorded for 74 h. Puromycin causing complete cell death at a concentration of 10 mg/mL (21 mM) was used as a control (*Fig. 7A*). Lower doses of **MB2Py(Ac)** (0.16, 0.8, and 4 μM) were found to have no effect on U2OS proliferation (*Fig. 7B*). At a concentration of 20 μM, a slowdown in proliferation was observed and 100-500 μM completely stopped cell division. It was demonstrated that **DB2Py(5) **inhibited osteosarcoma cells growth in a concentration- and time-dependent manner (*Fig. 7B*); i.e., the dimer was obviously more toxic than the monomer.

**MDR overcome**

An important property of a potential drug is its ability to overcome the MDR mediated by the ABC transporter of P-glycoprotein (P-gp). In this respect, the new compounds were tested by MTT in an immortalized, epithelial cell line HBL-100 [16, 17] and in a HBL-100/DOX subline obtained from HBL-100 by prolonged incubation with doxorubicin. It was shown that 95% of HBL-100/DOX cells overexpress the P-gp protein responsible for cell resistance to drugs, including cross-resistance to paclitaxel and vinblastine; in other words, the HBL-100/DOX subline had an MDR phenotype; i.e., it was resistant not only to doxorubicin, but also to other P-gp substrates [18].

The monomers demonstrated a similar cytotoxic effect in the HBL-100 line as in its stable subline; so, the differences in the *IC*50 values did not extend above 2-fold.

The data presented in *Table 2* show that the investigated bis-benzimidazole-pyrroles did not belong to the P-gp substrates. The HBL-100/DOX resistance to **DB2Py(4) **was 9-fold higher if compared to that to HBL-100, whereas the resistance of P-gpoverexpressing cells to such classical P-gp substrates as doxorubicin and paclitaxel increased 50–100 times and more. In this respect, a conclusion can be drawn that **DB2Py(4) **is a weak P-gp substrate; i.e., only the monomeric **MB2Py **and **MB2Py(Ac) **are able to completely overcome the MDR associated with P-gp overexpression.

**Cell-nucleus penetration**

**Fig. 8:**
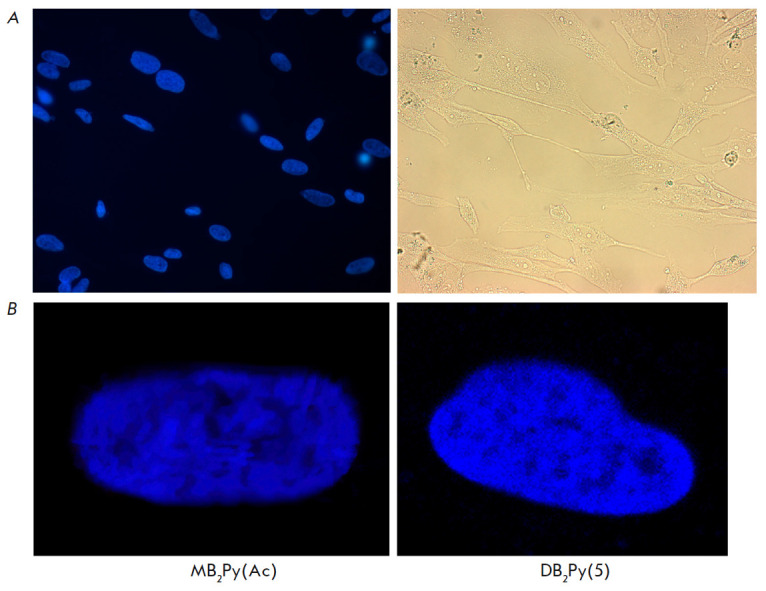
Fluorescent staining of Gbl13n glioblastoma cells incubated with monomeric and dimeric bis-benzimidazole-pyrroles at a concentration of 2 μM for 48 h. (*A*) – population of cells stained with the bis-benzimidazole- pyrrole** DB2Py(5)**. On the left is a DAPI filter, on the right is a phase-contrast image.** MB2Py(Ac) **staining looks similar after 2 days; (*B*) – a picture of stained nuclei obtained using a confocal microscope

How of the new compounds to penetrate within 2 days into the cell nucleus where they, binding to heterochromatin, glow in bright blue dots, was confirmed by fluorescence microscopy (*Fig. 8*).

Therefore, the synthesized compounds are new promising fluorescent dyes capable of penetrating cellular and nuclear membranes and effectively staining cell nuclei.

**Cell-cycle analysis**

**Fig. 9:**
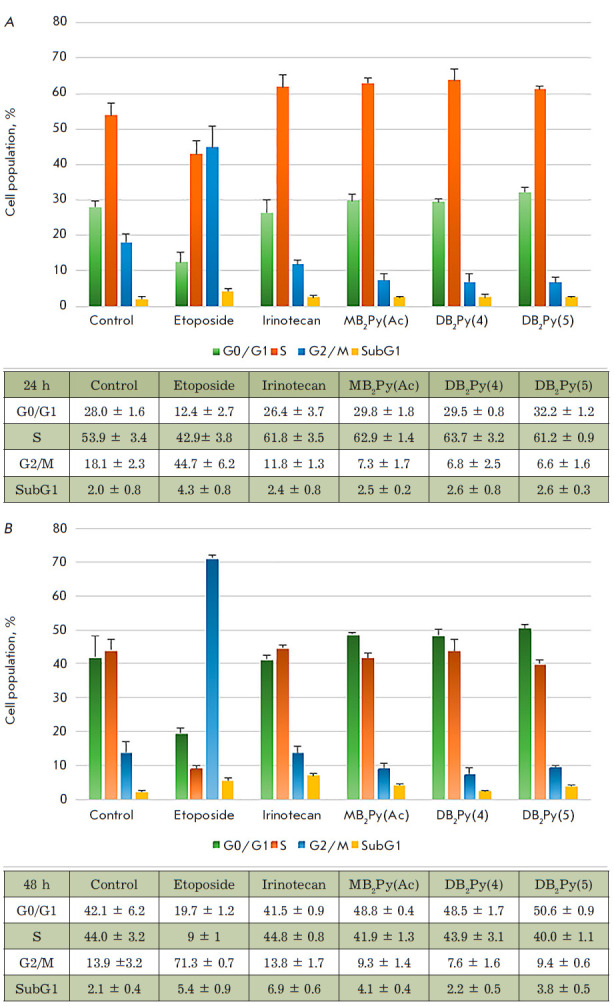
Effect of bis-benzimidazole- pyrroles on the cell cycle in an HCT-116 cell line. (*A*) – after a 24 h of incubation; (*B*) – after a 48 h of incubation

The way the new substances affect the cell cycle was investigated using two control drugs: etoposide and irinotecan. Etoposide stopped the cell cycle in mitosis as witnessed by the accumulation of cells in the G2/M phase and consistent with [19]. Forty-eight hours after etoposide treatment, the cell population in the G2/M phase had increased from 47 to 70%. Irinotecan arrested the cell cycle in the S-phase, leading to a reduced distribution of cell populations in other phases [20]. The cells exposed to irinotecan accumulated in the S-phase after 24 h and consequently in the G0/ G1 phase after 48 h. Apparently, the last accumulation was due to the cells that had time to divide and transit from mitosis to the G0/G1 phase. In 48 h, a slight increase in the proportion of cells in early apoptosis was observed (*Fig. 9*).

In 48 h, the cell populations redistributed towards an increase in the G0/G1 phase, proving that the new compounds affected the synthesis phase (S).

On the other hand, **DB2Py(4) **barely induced apoptosis. While the other substances induced early apoptosis, the values exceeded the control by only 2–3 times.

**Topo-I as a possible target for novel Hoechst 33258 derivatives**

**Table 2:** Cytotoxic activities of the novel bis-benzimidazole-pyrroles in the sensitive line HBL-100 and its resistant subline HBL-100/DOX with a MDR phenotype

Compound	HBL-100 HBL-100/DOX	Resilience	index^*^
	IC_50_ ± SD, μM		
Doxorubicin	0.6 ± 0.3	34 ± 6	57
MB_2_Py	58 ± 18	125 ± 21	2.1
MB_2_Py(Ac)	18 ± 11	29 ± 11	1.5
DB_2_Py(4)	4 ± 4.5	37 ± 11	8.9

^*^Resilience index is the ratio of IC_50_ in the stable subline HBL-100 and IC_50_ in the sensitive line HBL-100/DOX.

Some tumor types, such as breast, ovarian, and rectal cancers, are characterized by increased activity of Topo-I, an enzyme that plays a key role in cell function by regulating the DNA structure by its transcription, replication, recombination, and repair. Topo-I can relax (unwind) scDNA molecules by forming single-stranded breaks and then ligating them to relax supercoils. That capability currently makes Topo-I a recognized target for tumor targeting therapy [21, 22, 23].

DNA narrow-groove-binding ligands are able to compete with Topo-I for AT base-pair binding without covalently linking to DNA and significant changes in its conformation. In our study, Topo-I inhibition was detected through the ability of the tested compounds to delay the DNA relaxation reaction *in vitro*.

**Fig. 10:**
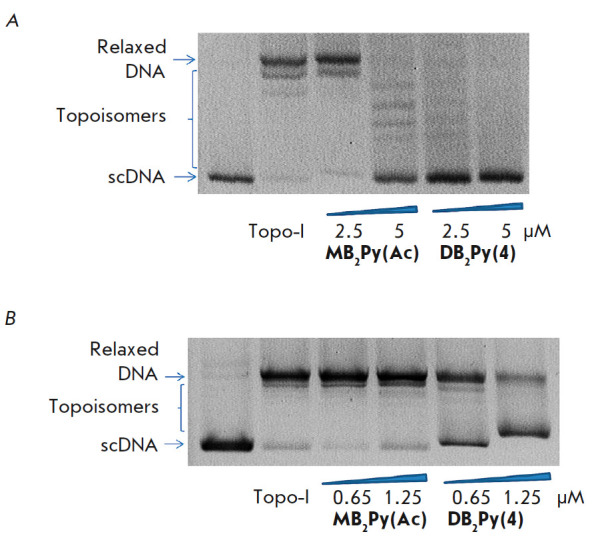
Calf-thymus Topo-I inhibition by **MB2Py(Ac) **and **DB2Py(4)**. (*A*) – effect of the compounds on Topo-I activity at concentrations of 2.5 and 5 μM. (*B*) – at concentrations of 0.65 and 1.25 μM

The studied compounds (*Fig. 10*) were found to inhibit Topo-I. At monomer and dimer concentrations of 5 and 2.5 μM, respectively, scDNA retention was observed in the DNA relaxation reaction (TopoGen) (*Fig. 10A*). Topo-I catalytic activity was most effectively inhibited by **DB2Py(4)**. If compared to **MB2Py(Ac)**, DNA retention was recorded at a** DB2Py(4) **concentration of just 0.65 μM (*Fig. 10B*).

## DISCUSSION

Benzimidazole is a fundamental pharmacophore in pharmaceutics because of its wide range of biological activities [24, 25, 26, 27]. When modeling new fluorescent bis-benzimidazole molecules, we hypothesized that the introduction of an AT-specific pyrrole carboxamide fragment with affinity for DNA AT pairs into previously obtained **DB(n)**, **DBA(n) **series would enhance their cytotoxicity. Two bis-benzimidazole units within the new molecule possessed fluorescent properties and interacted with DNA. A flexible linker in the** DB2Py(n) **dimers would allow the molecule to bind to two AT-rich sites located at various distances from each other. Binded with DNA, the new bis-benzimidazole- pyrroles had a planar shape isogeometric to the narrow DNA groove in order to enhance interaction between the new ligands and the DNA.

The tests employing absorption, fluorescence, and CD spectra demonstrated that the new compounds were able to interact with DNA. Since they had been derived from the **Hoechst 33258 **molecule known for its localization in a narrow DNA groove, we classified them as DNA narrow-groove-binding ligands [28, 29]. The presence of two bis-benzimidazole fragments in the ligand molecule leads to a significant increase in its affinity towards polynucleotide, which provides an experimental basis for the targeted synthesis of a new class of potential antitumor drugs based on dimeric bis-benzimidazoles.

The new fluorescent compounds have shown their ability to influence the S-phase of the cell cycle; to penetrate into the cell nucleus, and to inhibit Topo-I at low concentrations in a cell-free model. The new series of bis-benzimidazole-pyrroles has turned out to be more toxic against human tumor cell lines than the previously obtained **DB(n) **and **DBA(n) **series and less toxic to a cell line of non-tumor nature. Small (2-fold) but statistically significant differences in cytotoxicity have been demonstrated in a pair (tumorigenic and non-tumorigenic) of human breast cell lines.

Our earlier studies showed that bis-benzimidazolepyrroles were able to induce Bcl-xl-mediated apoptosis [30]. Since netropsin is known to affect the activity of eukaryotic transcription factors [31, 32], we assumed that the new compounds containing a netropsin fragment in their structure would have a similar action mechanism, which is supported by the data on DNA binding and cell cycle arrest in the synthesis phase at non-toxic concentrations of bis-benzimidazole- pyrroles.

Dimerization of the molecule enhances its affinity to DNA and Topo-I inhibitory properties *in vitro*. However, a MTT analysis of the cytotoxicity of the new compounds in tumor-cell lines did not reveal a clear advantage for the dimeric molecule, despite its ability to penetrate the cell nucleus. Nevertheless, a highly sensitive, real-time proliferation test confirmed the enhanced toxic properties upon bis-benzimidazolepyrrole dimerization.

The important characteristics of the new compounds as potential antitumor agents are their selectivity and ability to overcome MDR. One of the main reasons for the poor efficacy of modern chemotherapy is the selection of tumor cells with a MDR phenotype that can survive lengthy drug administration. The best known MDR mechanism is overexpression of the P-gp protein, a member of the ABC transporter family. Preliminary detection of bis-benzimidazole-pyrrole cytotoxicity in a cell model with a MDR phenotype showed that dimerization of the molecule may have led to an interaction with the P-gp protein and, as a consequence, to an increase in the resistance of the HBL-100/DOX line to this compound (9-fold on average) compared to doxorubicin (50–100-fold or more); so, it would be interesting to investigate the possibility of direct interaction between the new molecules and the P-gp transporter.

Perhaps the reason why dimeric compounds remain underestimated in terms of their biological activity is their greater tendency to form aggregates as compared to monomeric molecules. Finding a way to overcome the aggregation of dimeric molecules can pave the way to designing significantly more active compounds.

## CONCLUSION

The newly synthesized cytotoxic dimeric bis-benzimidazole- pyrroles appear promising for further indepth study of their properties and action mechanism against human tumor cells, as well as for designing new molecules.

## Abbreviations

DMF: dimethylformamide
MDR: multidrug resistance
Topo-I: eukaryotic topoisomerase I
CLCD: cholesteric liquid crystal dispersion
IC50: 50% inhibitory concentration
